# Binding of Soluble Yeast β-Glucan to Human Neutrophils and Monocytes is Complement-Dependent

**DOI:** 10.3389/fimmu.2013.00230

**Published:** 2013-08-12

**Authors:** Nandita Bose, Anissa S. H. Chan, Faimola Guerrero, Carolyn M. Maristany, Xiaohong Qiu, Richard M. Walsh, Kathleen E. Ertelt, Adria Bykowski Jonas, Keith B. Gorden, Christine M. Dudney, Lindsay R. Wurst, Michael E. Danielson, Natalie Elmasry, Andrew S. Magee, Myra L. Patchen, John P. Vasilakos

**Affiliations:** ^1^Biothera, Eagan, MN, USA

**Keywords:** C3, opsonization, CR3, β-glucans, neutrophils, monocytes

## Abstract

The immunomodulatory properties of yeast β-1,3/1,6 glucans are mediated through their ability to be recognized by human innate immune cells. While several studies have investigated binding of opsonized and unopsonized particulate β-glucans to human immune cells mainly via complement receptor 3 (CR3) or Dectin-1, few have focused on understanding the binding characteristics of soluble β-glucans. Using a well-characterized, pharmaceutical-grade, soluble yeast β-glucan, this study evaluated and characterized the binding of soluble β-glucan to human neutrophils and monocytes. The results demonstrated that soluble β-glucan bound to both human neutrophils and monocytes in a concentration-dependent and receptor-specific manner. Antibodies blocking the CD11b and CD18 chains of CR3 significantly inhibited binding to both cell types, establishing CR3 as the key receptor recognizing the soluble β-glucan in these cells. Binding of soluble β-glucan to human neutrophils and monocytes required serum and was also dependent on incubation time and temperature, strongly suggesting that binding was complement-mediated. Indeed, binding was reduced in heat-inactivated serum, or in serum treated with methylamine or in serum reacted with the C3-specific inhibitor compstatin. Opsonization of soluble β-glucan was demonstrated by detection of iC3b, the complement opsonin on β-glucan-bound cells, as well as by the direct binding of iC3b to β-glucan in the absence of cells. Binding of β-glucan to cells was partially inhibited by blockade of the alternative pathway of complement, suggesting that the C3 activation amplification step mediated by this pathway also contributed to binding.

## Introduction

Yeast β-glucans are represented in various forms such as intact yeast, zymosan, purified whole glucan particle, solubilized zymosan polysaccharide, or highly purified soluble β-glucans of different molecular weights (1–11). Structurally, yeast β-glucans are mainly composed of glucose monomers organized as a β-(1-3)-linked glucopyranose backbone with periodic β-(1-3) glucopyranose branches linked to the backbone via β-(1-6) glycosidic linkages. Studies of the mechanisms through which the yeast β-glucans exert their immunomodulatory effects have largely been focused on evaluation of the most basic and simple structural differences between β-glucans, such as their particulate or soluble nature, to the more complex structural characteristics that determine the tertiary conformation including, length of the main chain, length of the side chains, and frequency of the side chains.

Yeast β-glucans are fungal pathogen associated molecular patterns (PAMPs) and are recognized by pattern recognition receptors on cellular membranes as well as pattern recognition molecules in the serum. Complement receptor 3 (CR3, CD11b/CD18, α_M_β_2_-integrin, Mac-1) and Dectin-1 have been reported to be the predominant cell surface pattern recognition receptors for yeast β-glucans on innate immune cells including, monocytes, macrophages, dendritic cells, and neutrophils (1–9). Several studies have shown that both particulate β-glucans as well as various forms of yeast-derived soluble β-glucans bind to both CR3 and Dectin-1 (1, 2, 4, 12–16). Soluble zymosan polysaccharide (SZP), rich in mannan or β-glucan (∼10 kDa), neutral soluble glucan (∼25 kDa, NSG), a chemically modified soluble yeast β-glucan (∼127 kDa, glucan phosphate) have all been shown to bind to CR3 on human peripheral blood isolated neutrophils (7, 8). Of these, glucan phosphate and NSG have also been used as antagonists of Dectin-1 receptor (3, 4, 17, 18). The highly purified soluble β-(1,6)-[poly-(1,3)-d-glucopyranosyl]-poly-β(1,3)-d-glucopyranose (PGG) glucan (∼120–205 kDa) has also been demonstrated to bind to recombinant human Dectin-1 and signal via CR3 on human neutrophils (17, 19).

With respect to pattern recognition molecules in serum, complement proteins can recognize pathogens and subsequently be activated by the classical, alternative, or lectin pathways (20, 21). All three culminate in activation of C3, the central step in complement activation, which is then followed in the final steps by formation of the cytolytic membrane attack complex (MAC). After the initial C3 proteolytic activation step, nascently activated C3b can covalently associate with the carbohydrates or proteins present on the surface of the pathogen. This initial process of C3b attachment to pathogens is followed by further inactivation of bound C3b by its proteolysis to iC3b, followed by further degradation to C3d (20, 21). Complement opsonization of pathogens can lead to either direct killing of the pathogen by formation of MAC or recognition and destruction of the C3 fragment-opsonized pathogen by cell-associated complement receptors, CR1, CR2, CR3, or CR4 on leukocytes. Zymosan, a crude particulate β-glucan obtained from cell walls of *Saccharomyces cerevisiae* is well known as a stimulator of the antibody-independent alternative pathway of complement activation (22–25). β-glucan from *Candida albicans*, a pathogenic fungus has also been shown to activate the alternative pathway of complement (26). Some studies have also shown a role for both the classical and the alternative pathways in opsonization of zymosan and glucan from the fungi *Cryptococcus neoformans* and *Blastomyces dermatitidis* (27–31). Curdlan, a linear β-1,3 glucan coupled to a resin has been demonstrated to be recognized by MBL and l-ficolin in human serum and to activate the lectin pathway of complement activation (32).

In binding studies to date, both complement opsonized and unopsonized fungus, and particulate β-glucans have been demonstrated to bind to CR3, CR4, and Dectin-1 (1, 2, 4, 12–16, 33). However, the role of complement opsonization in binding of soluble β-glucan to CR3 or Dectin-1 has not been studied. In this report we have investigated the binding of *Saccharomyces cerevisiae*-derived, highly purified, well-characterized soluble β-glucan, PGG β-glucan, to human monocytes and neutrophils, the innate immune cells expressing the reported β-glucan receptors. The results confirm some of the earlier findings that binding of soluble yeast β-glucan to human monocytes and neutrophils is CR3-mediated. In addition, we also demonstrate that the soluble β-glucan binds to CR3 in a complement-dependent manner. Complement opsonization of the soluble β-glucan was shown to be a critical requirement for its binding to the immune cells.

## Materials and Methods

### Antibodies and reagents

The list of antibodies (Abs) used in the study as well as their source and specificities are shown in Table 1. Antibody-sensitized sheep erythrocytes (EA), MicroVue SC5b-9 Plus EIA kit, and MicroVue C4a EIA kit were from Quidel (San Diego, CA, USA). Compstatin (ICVVQDWGHHRCT) and control peptide (IAVVQDWGHHRAT) were from Tocris Bioscience (Bristol, UK). Dextran, ethylene glycol tetraacetic acid (EGTA), magnesium chloride (MgCl_2_), and 4′,6-diamidino-2-phenylindole (DAPI) were from Sigma Aldrich (St. Louis, MO, USA). Pyrogene™ endotoxin kit was purchased from Lonza (Walkersville, MD, USA).

**Table 1:** **Description of antibodies used in the study**.

Designation	Isotype	Specificity	Source
HI111	Murine IgG1 mAb	Human CD11a	eBioscience (San Diego, CA)
LM2/1	Murine IgG1 mAb	Human CD11b (I-domin)	eBioscience
VIM12	Murine IgG1 mAb	Human CD11b (Lectin-like domain)	Invitrogen (Camarillo, CA)
IB4	Murine IgG2a mAb	Human CD18	Ancell (Bayport, MN)
GE2	Murine IgG1 mAb	Human Dectin-1	AbD Serotec (Raleigh, NC)
iC3b	Murine IgG2b mAb	Human iC3b (neo antigen)	Quidel (San Diego, CA)
166–32	Murine IgG1 mAb	Human factor D	ATCC (Manassas, VA)
BfD IV	Murine IgM mAb	Yeast β-1,3/1,6-glucan	Biothera (Eagan, MN)
CD14	Murine IgG1 mAb	Human CD14	BioLegend (San Diego, CA)
CD15	Murine IgG1 mAb	Human CD15	BioLegend
IgG1	Murine IgG1	Isotype control	eBioscience
IgG2a	Murine IgG2a	Isotype control	eBioscience
FITC-conjugated F(ab′)_2_ Goat anti-Mouse IgM	Goat polyclonal	Mouse IgM	Jackson ImmunoResearch Lab (West Grove, PA)
Cy3-conjugated Goat anti-mouse IgG	Goat polyclonal	Mouse IgG	BioLegend
Cy3-conjugated Goat anti-mouse IgM	Goat polyclonal	Mouse IgM	Jackson ImmunoResearch Lab

### Preparation and characterization of β-glucan

PGG β-glucan, a pharmaceutical-grade soluble yeast 1,3/1,6 β-glucan was manufactured from a strain of *Saccharomyces cerevisiae* generated by Biothera (Eagan, MN, USA). As part of the manufacturing and quality control process, PGG β-glucan was extensively characterized analytically with respect to the parameters listed in Table 2. For some experiments, PGG β-glucan was prepared for use by performing a buffer exchange into Dulbecco’s phosphate-buffered saline (DPBS) using 3 kDa molecular weight cut-off (MWCO) Amicon centrifugal filtration units (Millipore, Billerica, MA, USA). The hexose concentration of the β-glucan preparations was determined by the anthrone method (34). Preparation of particulate β-glucan has been described previously (35, 36).

**Table 2:** **Analytical characterization of PGG β-glucan**.

Parameters	Results
Average molecular weight^a^	150,000 Da
Branching^b^	4.1%
Purity^c^	
Residual Protein^d^	≤0.2%
% Mannans (mannose)^e^	≤0.6%
% Glycogen^f^	≤5%
% Chitin (glucosamine)^g^	≤0.4%

*^a^Gel permeation chromatography (GPC) with differential refractive index (dRI) and multi angle laser light scattering (MALLS) detection*.

*^b^Partially methylated alditol acetate method using gas chromatography with flame ionization detection*.

*^c^Expressed as % of total hexose*.

*^d^Detection by Bradford protein assay*.

*^e^Hydrolysis with trifluoroacetic acid to monosaccharides followed by high performance anion exchange chromatography with pulsed amperometric detection*.

*^f^Digestion by amyloglucosidase and detection of liberated glucose by an enzymatic glucose detection assay*.

*^g^Hydrolysis with sulfuric acid followed by reaction with acetyl acetone and Ehrlich’s reagent and detection spectrophotometrically*.

### Isolation of human peripheral blood mononuclear cells and neutrophils

Heparinized venous blood was obtained from healthy individuals with informed consent as approved by the Institutional Review Board (approved by the New England Institutional Review Board, Wellesley, MA, USA, Blood Donation Protocol No. 07-124). Briefly, peripheral blood mononuclear cells (PBMC) were isolated by Ficoll-Paque (Amersham Biosciences, Piscataway, NJ, USA) density gradient centrifugation. Neutrophils were subsequently enriched by sedimentation with 3% dextran, followed by hypotonic lysis of residual erythrocytes. The purity and viability of neutrophils and PBMC obtained were consistently>95%.

### Preparation of human autologous serum

Human serum was prepared according to vendor’s instruction. Ten milliliters of non-heparinized whole blood (WB) was added to a Vacutainer^®^ SSTTM tube (Becton Dickinson, NJ, USA) and inverted 3–5 times. The blood was allowed to clot by incubation at room temperature for 30 min, and then the sample was centrifuged at 2000 rpm (∼1150 × *g*) for 10 min and cleared serum was collected and used as needed. For preparing heat-inactivated (HI) serum, the serum was incubated in a 56°C water bath for 30 min. All binding experiments were performed using autologous serum. In this study, autologous (complement-intact) human serum will be hereafter referred to as serum and after heating, as HI serum.

### PGG β-glucan binding studies

Enriched neutrophils or PBMC were resuspended at 1 × 10^6^ cells/milliliters in RPMI 1640 supplemented with 10% human serum. In dose-titration studies, PGG β-glucan at hexose concentrations 10, 25, 100, 200, or 400 μg/mL were added to neutrophils or PBMC and incubated in a 37°C, 5% CO_2_ humidified incubator for 1 h. In subsequent experiments, PGG β-glucan was added at 100 μg/mL to both neutrophils and PBMC. After incubation, cells were washed twice with FACS buffer (HBSS supplemented with 1% fetal bovine serum and 0.1% sodium azide) to remove any unbound β-glucan, and subsequently treated with Fc block (Miltenyi Biotec, Auburn, CA, USA). After the Fc block step, cells were stained with the BfD IV mouse IgM mAb for 30 min at 4°C and washed twice with cold FACS buffer. Cells were then incubated with FITC-conjugated F(ab′)_2_ goat anti-mouse IgM for 30 min at 4°C and washed once with cold FACS buffer before fixing with 1% paraformaldehyde. The generation and specificity of Biothera-produced β-1,3/1,6-glucan-specific mAb BfD IV (mouse IgM, clone 10C6) has been described previously (37). In certain optimization experiments, neutrophils and monocytes were identified by staining with fluorescently labeled anti-CD15, or anti-CD14 Abs respectively. Events were collected on a LSRII flow cytometer (BD Biosciences, San Jose, CA, USA) and analysis was performed using FlowJo (Tree Star, Ashland, OR, USA).

### CR3 and Dectin-1 binding studies

To evaluate the role of CR3 or Dectin-1 receptors in binding, the cells were pre-incubated with specific receptor blocking Abs or the relevant isotype controls at 4°C for 30–45 min before addition of 100 μg/mL PGG β-glucan and measurement of binding was performed as described earlier. The CR3 blocking Abs used were LM2/1, a mouse anti-human IgG1 monoclonal antibody to the I-domain of the CD11b chain of CR3, VIM12, a mouse monoclonal IgG1 anti-human antibody to the lectin domain of the CD11b chain of CR3, and IB4, a mouse monoclonal IgG2a anti-human antibody to the CD18 chain of CR3. Each blocking Ab was used at 10 μg/1 × 10^6^ cells. Combinations of CR3 blocking Abs used were either LM2/1 + VIM12 to block both the I-domain and lectin-domains of the CD11b subunit or LM2/1+ VIM12 + IB4 to block both the CD11b and CD18 subunits of CR3. HI111, a mouse monoclonal IgG1 anti-human antibody to an irrelevant integrin, the CD11a chain of LFA-1 was used at 10 μg/mL as a negative control in some of the blocking experiments. For blocking the Dectin-1 receptor, clone GE2, a mouse monoclonal IgG1 anti-human antibody was used at 10 μg/mL. All the isotype controls were used at the same concentration as the blocking Abs.

### Binding studies to determine serum, time, and temperature dependency

For these experiments, PGG β-glucan was used at 100 μg/mL and binding determined as described above with the following changes. For determining serum dependency, PGG β-glucan was incubated with cells resuspended in RPMI 1640 containing either 2, 5, 10, 20, or 50% serum at 37°C for 1 h. For kinetic experiments, PGG β-glucan was incubated with cells resuspended in 10% serum at 37°C for 10, 30, 60, or 120 min. For temperature dependency experiments, PGG β-glucan was incubated with cells resuspended in 10% serum for 1 h at 4°C, room temperature, or 37°C.

### Binding of PGG β-glucan after pre-opsonization

Here pre-opsonization of β-glucan is defined as the process of incubating PGG β-glucan in serum before adding the β-glucan to neutrophil or PBMC cultures containing serum. One part PGG β-glucan was added to nine parts serum at a final β-glucan concentration of 6–8 mg/mL and incubated for 30 min at 37°C in a water bath. The serum-pretreated β-glucan (OpPGG) was then added to cells to obtain a final concentration of 100 μg/mL. At this concentration, the final concentration of serum transferred into the cell cultures amounted to less than 2%. The ability of OpPGG to bind to the cells at 37°C, after resuspension in 10% serum or in HI serum was subsequently measured.

### Binding studies to determine the role of serum complement proteins

To determine the role of serum complement in binding of PGG β-glucan, experiments were performed as described above except for variation to the serum conditions. Serum conditions used in these studies included 10% HI serum, compstatin-treated serum, EGTA + MgCl_2_ (MgEGTA)-treated serum, or factor D-blocked serum. For compstatin- and control peptide-treated serum, compstatin or the control peptide was added to the serum at 20 or 100 μM and incubated at room temperature for 10 min. For blocking of factor D in the serum, 166–32 mAb at a concentration of 20 μg/mL was incubated with serum on ice for 30 min, and then used for binding studies. For binding experiments in the presence of MgEGTA, serum was incubated with 10 mM EGTA with 10 mM MgCl_2_ for 10 min at room temperature. The untreated and MgEGTA-treated serum was then used to pre-opsonize PGG β-glucan and perform binding studies as described above.

### iC3b staining on PGG β-glucan-bound cells

Binding of PGG β-glucan to neutrophils and PBMC was performed as described above. iC3b deposition on these cells was detected by staining with a neo-epitope specific anti-iC3b mAb and PE-conjugated goat anti-mouse IgG followed by flow cytometry analysis.

### ELISA for iC3b deposition on immobilized PGG β-glucan

PGG β-glucan or dextran were immobilized on wells of a 96 well polystyrene Costar^®^plate (Corning, NY, USA) by drying at 50°C followed by ultra-violet cross-linking. The coated plates were first blocked with 1% BSA before incubation with the untreated serum or serum that had undergone various treatments as described in binding studies. For this step, the serum was diluted 1:2 with wash buffer (PBS/0.05% Tween-20) and plates were incubated for 30 min at 37°C. Each treatment condition was performed in triplicate. After washing off the serum from the plate, bound iC3b was detected by using the anti-iC3b mAb followed by biotin-labeled goat anti-mouse IgG Ab. Binding of the biotin-labeled antibody was determined using streptavidin peroxidase and 3,3′,5,5′-Tetramethylbenzidine substrate solution (KPL, Gaithersburg, MD, USA). Optical density at 450 nm (OD_450_) of each well was measured with a SpectraMAX 250 (Molecular Devices, CA, USA). The fold change was calculated by dividing the OD_450_ of wells containing immobilized PGG β-glucan or dextran by the OD_450_ of blank wells, incubated in the presence of treated or untreated serum.

### Immunoprecipitation of PGG β-glucan

One part PGG β-glucan was added to nine parts serum or HI serum at a final β-glucan concentration of 6–8 mg/mL and incubated for 30 min at 37°C in a water bath. BfD IV mAb was added to the serum-glucan mixture and incubated at room temperature for an additional 30 min. Magnetic beads conjugated with rat anti-mouse IgM (Dynabeads) were washed three times with DPBS and incubated with the serum-PGG β-glucan/dextran-BfD IV mixture for another 30 min at room temperature. The beads were separated magnetically and any iC3b that was pulled-down along with the immunoprecipitated PGG β-glucan was detected by flow cytometry using FITC-conjugated iC3b mAb.

### Measurement of fluid-phase SC5b-9 complex formation in the serum

The MicroVue SC5b-9 EIA kit was used to measure activation of the classical and alternative pathways of complement according to the vendor’s instruction (Quidel). Briefly, the serum (untreated or various treated serum preparations) was mixed with PBS, EA, or 20 units of cobra venom factor (CVF) and added to the plate wells pre-coated with anti-SC5b-9 mAb. The plate was incubated at 37°C for 60 min followed by five washes with the provided wash buffer. The plate was then incubated at room temperature for 30 min with the provided SC5b-9 Plus Conjugate that contained a horseradish peroxidase-conjugated Ab specific for SC5b-9. The plate was then washed five times, incubated with the substrate (see above) for 15 min at room temperature to initiate the enzymatic reaction and subsequently quenched with the stop solution. OD_450_ was measured. The concentration of fluid-phase SC5b-9 present in the samples was determined from the standard curve generated with the provided SC5b-9 standards.

### Measurement of C4a levels

The MicroVue C4a EIA kit to measure C4a levels in the plasma was used according to the vendor’s instruction (Quidel). Briefly, WB was either treated with vehicle, or, 10 μg/mL PGG β-glucan, or 10 μg/mL particulate β-glucan at 37°C for 30 min. After stimulation, WB was spun for 5 min at 2000 rpm and cell-free plasma was collected. Plasma samples were added to the plate wells pre-coated with anti-C4a mAb. The plate was incubated at 37°C for 60 min followed by five washes with the provided wash buffer. The plate was then incubated at room temperature for 60 min with the provided Conjugate reagent containing a horseradish peroxidase-conjugated Ab specific for C4a. The plate was then washed five times, incubated with the substrate reagent for 15 min at room temperature to initiate the enzymatic reaction, and subsequently quenched with the stop solution. OD_450_ was measured. The concentration of C4a present in the samples was determined from the standard curve generated with the provided C4a standards.

### Confocal microscopy

Enriched neutrophils resuspended at 1 × 10^6^ cells/milliliters in RPMI supplemented with 20% serum or HI serum were mixed with PGG β-glucan before applying onto 10 mm glass cover slips placed in the wells of a 24 well plate. The plate was incubated in a 37°C, humidified 5% CO_2_ incubator for 1 h. Unbound cells and PGG β-glucan were removed by washing with warmed PBS, and bound cells were subsequently fixed in 1% paraformaldehyde at room temp for 15 min. Cells were blocked with Fc block prior to staining with BFD IV and anti-iC3b mAb for 30 min at 4°C. The cells were then stained by secondary Abs, Cy5-conjugated goat anti-mouse IgM and Cy3-conjugated goat anti-mouse IgG at 4°C for 30 min. Cells were permeabilized with 0.1% ice-cold TritonX-100 for 3 min on ice and stained with DAPI on ice for 5 min before mounting onto slides. Images were analyzed and acquired with an Olympus FluoView 1000 IX2 Inverted confocal microscope. Images were adjusted equally in Adobe Photoshop (Adobe Systems Inc., San Jose, CA, USA).

### Data analysis

The neutrophils and monocytes were assessed for their capacity to bind PGG β-glucan by comparing the mean fluorescence intensity (MFI) of the cells stained with the anti-β-glucan antibody, BfD IV and the percentage of cells positive for BfD IV relative to values obtained in vehicle-treated control group. For inhibition of binding studies, Percent inhibition of Imprime PGG binding is calculated based on MFI values of BfD IV positive cells in the presence and absence of blocking Abs. The formula used for calculating percent inhibition is:

$$\frac{\begin{array}{c} \left(\mathrm{MF}I_{\mathrm{PGG}-\mathrm{treated}\;\mathrm{group}\;\mathrm{with}\;\mathrm{controls}-\mathrm{MF}I_{\mathrm{vehicle}}}\right)-\\ \left(\mathrm{MF}I_{\mathrm{PGG}-\mathrm{treated}\;\mathrm{group}\;\mathrm{with}\;\mathrm{inhibitory}\;\mathrm{agents}-\mathrm{MF}I_{\mathrm{vehicle}}}\right) \end{array}}{\left(\mathrm{MF}I_{\mathrm{PGG}-\mathrm{treated}\;\mathrm{group}\;\mathrm{with}\;\mathrm{controls}-\mathrm{MF}I_{\mathrm{vehicle}}}\right)}$$

Statistical analysis to compare different treatment groups to each other were done by performing Student *t*-test; *p* ≤ 0.05 was considered significant.

## Results

### PGG β-glucan binds to human neutrophils and monocytes in a CR3-dependent manner

**Figure 1:**
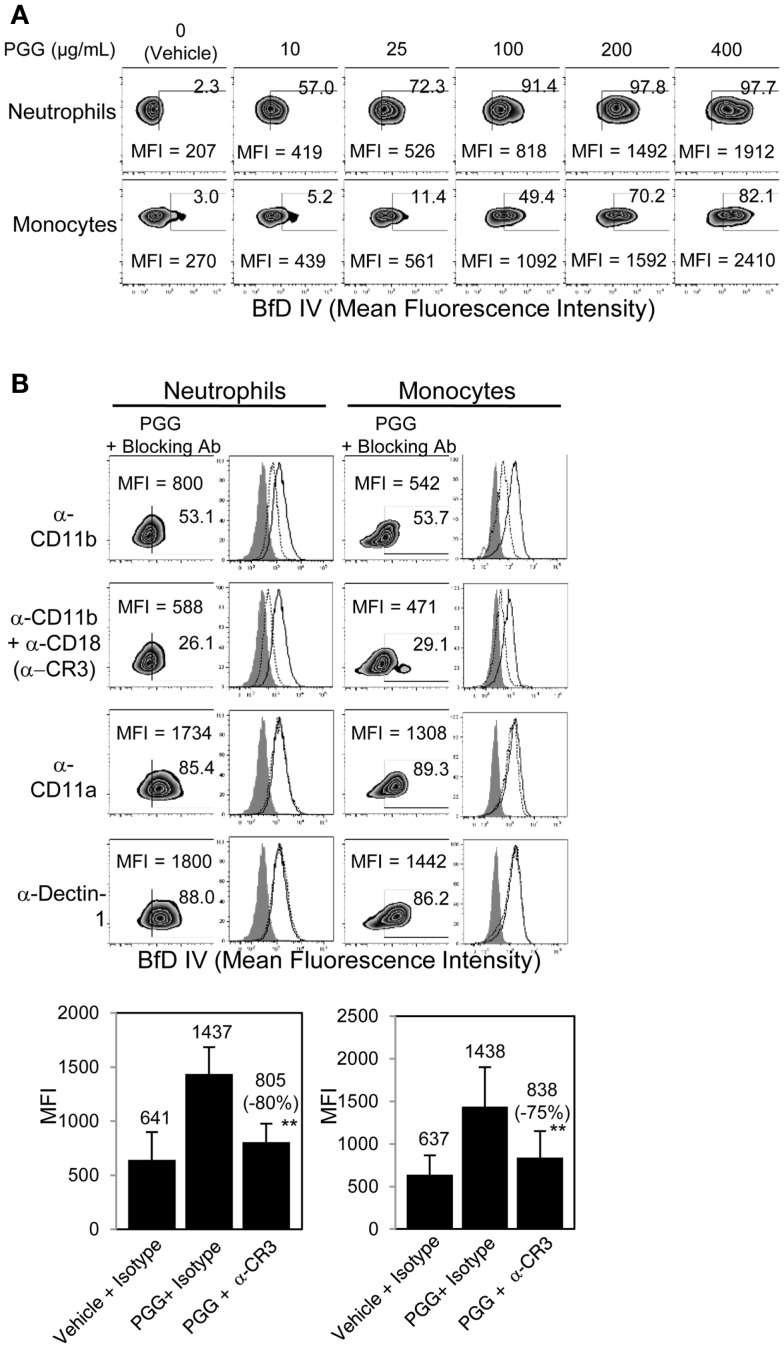
**Role of CR3 receptor in binding of PGG β-glucan to human neutrophils and monocytes**. **(A)** Binding of increasing concentration of PGG β-glucan (0, 10, 25, 100, 200, and 400 μg/mL) to human neutrophils (upper row) and monocytes in PBMC (lower row) was measured by flow cytometry after incubation of cells with β-glucan or vehicle at 37°C for 1 h. **(B)** To identify the receptor involved, binding of PGG β-glucan to human neutrophils (left) and monocytes (right) was measured in the presence of α-CD11b (upper row), α-CR3 (second row), α-CD11a (third row), or α-Dectin-1 blocking antibodies (bottom row). The MFI and percentage of β-glucan-treated, BfD IV positive cells are indicated in the zebra plots. Histograms show PGG β-glucan binding in the presence of isotype controls (solid) and blocking antibodies (dotted) compared to vehicle-control binding (gray filled). Bars represent the mean MFI of the vehicle- or PGG β-glucan-treated groups in the presence of CR3 blocking or isotype control antibodies from five donors. The percentage of inhibition (in parentheses) was indicated on the graph. **p ≤ 0.05 compared to β-glucan-treated group in the presence of isotype controls. Data shown are representative of at least three independent experiments performed with cells from different donors.

The binding of PGG β-glucan to human neutrophils and monocytes was evaluated by incubating 10, 25, 100, 200, and 400 μg/mL of PGG β-glucan or vehicle with the cells resuspended in media with 10% serum, and then staining the cells with a β-glucan-specific mAb, BfD IV plus a fluorophore labeled secondary antibody. These concentrations were chosen to evaluate *in vitro* binding of PGG β-glucan at concentrations on both the lower and higher side of the maximum concentration achieved in the serum (Cmax) of healthy volunteers and cancer patients administered PGG β-glucan. To date, the range of Cmax values observed in healthy volunteers is 35.49–66.5 μg/mL with the average being 51.24 ± 15.45 μg/mL, while in cancer patients, this range is 18.3–62.4, with the average being 39.5 ± 19.2 μg/mL (unpublished data from clinical trials NCT00542217 and NCT00545545). As shown in Figure 1A, PGG β-glucan at concentrations bound to both neutrophils and monocytes in a dose-dependent manner. While for both cell types, the percentage of BfD IV positive cells reached a plateau at 200 μg/mL, the MFI values continued to increase with increasing concentrations of PGG β-glucan. The variability in the extent of PGG β-glucan binding to the neutrophils and monocytes in multiple donors as demonstrated by the variability in the MFI values achieved is shown in Figure S1 in Supplementary Material.

In order to determine the role of CR3 or Dectin-1 in the binding of PGG β-glucan to human neutrophils and monocytes, these receptors were blocked with receptor-specific blocking Abs or irrelevant control Abs before addition of PGG β-glucan and measurement of binding. Data presented in Figure 1B shows that blocking the CD11b chain (LM2/1 + VIM 12) of CR3 partially inhibited binding of PGG β-glucan to both neutrophils and monocytes, while blocking both the CD11b and CD18 chains (LM2/1 + VIM12 + IB4) of CR3 further inhibited PGG β-glucan binding. Blocking the alpha chain of a non-specific integrin, CD11a chain of LFA-1, did not affect PGG β-glucan binding. Moreover, blocking the other major β-glucan receptor, Dectin-1 (GE2), did not inhibit PGG β-glucan binding to neutrophils or monocytes. Based on results from five different donors, the average MFI of BfD IV positive cells treated with CR3 blocking Abs (LM2/1 + VIM12 + IB4) was significantly lower than that of the BfD IV positive cells treated with the isotype control Abs. Based on the MFI values in these five donors, a percentage of inhibition of binding by the CR3 blocking Abs was calculated relative to that of the isotype control Abs in these five donors. The range of percentage of inhibition of binding by blocking both of the CR3 chains in neutrophils was 69–100% with an average inhibition of 80%, and in monocytes the range was 42–95% with an average of 75%. Overall, the results demonstrate that in the presence of serum, CR3 plays a major role as a receptor involved in the binding of PGG β-glucan to human neutrophils and monocytes.

### Binding of PGG β-glucan to human neutrophils and monocytes is serum-, time-, and temperature-dependent

**Figure 2:**
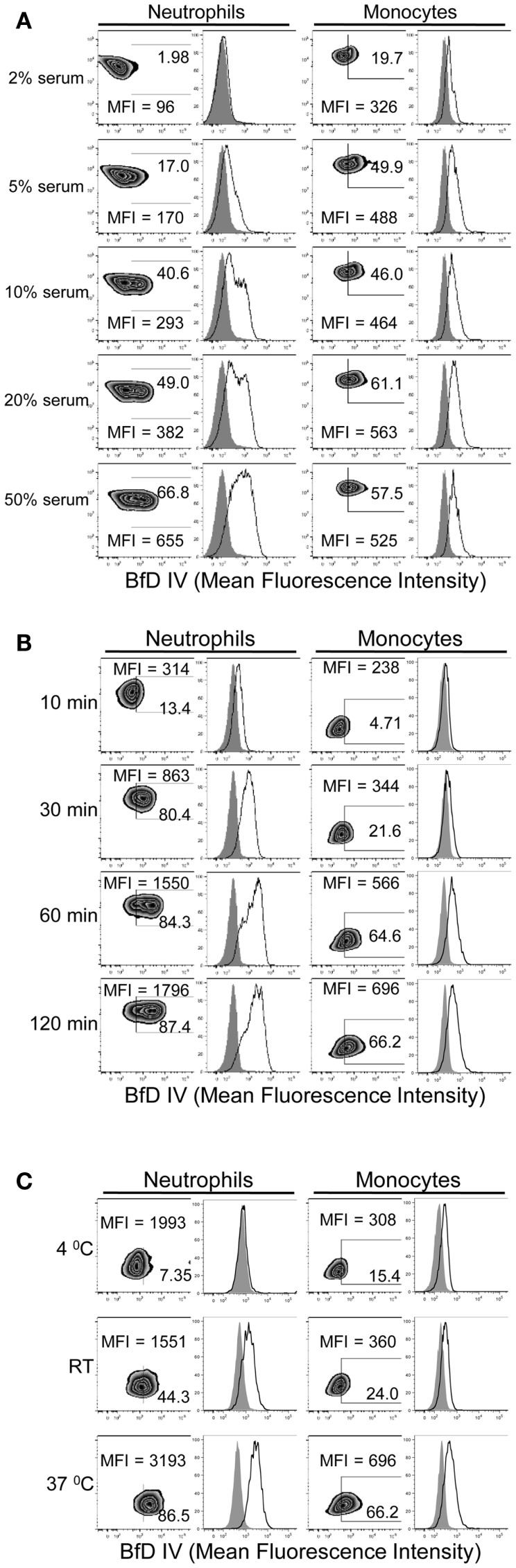
**Time, temperature, and serum-dependent binding of PGG β-glucan to human neutrophils and monocytes**. PGG β-glucan binding to human neutrophils and monocytes was determined **(A)** in the presence of 2, 5, 10, 20, and 50% of serum, **(B)** at 10, 30, 60, and 120 min, and **(C)** at RT, 4 and 37°C. The MFI and percentage of BfD IV positive cells were indicated in the zebra plots, and histogram shows PGG β-glucan binding (solid) in comparison to the vehicle-control (gray filled). Data shown in each of the conditions are representative of three independent experiments performed with cells obtained from three different donors.

In order to determine the conditions required for PGG β-glucan binding to human neutrophils and monocytes, the influence of serum, time, and temperature were evaluated. The role of serum in PGG β-glucan binding to cells was evaluated after incubating cells at 37°C for 1 h in media with 2, 5, 10, 20, or 50% serum. The data in Figure 2A demonstrate that binding of PGG β-glucan increased proportionally with the percentage of serum present in the media. At the tested serum concentrations, minimum and maximal binding occurred on neutrophils at 2 and 50% serum, respectively. However, for monocytes, the maximum binding was observed at 10% serum, while a reduction in binding was seen at 20 and 50% serum. The variability in binding of PGG glucan at the different serum concentrations in multiple donors as demonstrated by the variability in the MFI achieved is demonstrated in Figure S2 in Supplementary Material. The effects of incubation time and temperature on PGG β-glucan binding were evaluated under conditions using media containing 10% serum. In kinetic experiments, binding was measured at 10, 30, 60, and 120 min of incubation at 37°C. To evaluate the influence of temperature, binding was measured after cells were incubated with PGG β-glucan for 1 h at 4°C, room temperature, or 37°C. The results presented in Figure 2B demonstrate that PGG β-glucan binding increases with incubation time. For neutrophils, optimal binding occurred at 30–60 min, while 60–120 min were required for optimal binding to monocytes. Temperature also affected the binding of PGG β-glucan to cells; optimal binding occurred when PGG β-glucan was incubated with cells at 37°C as compared to 4°C or room temperature (Figure 2C). These data demonstrate that binding of PGG β-glucan to human neutrophils and monocytes is serum-, time-, and temperature-dependent.

### Binding of soluble PGG β-glucan to human neutrophils and monocytes is dependent on complement

After evaluating the importance of serum in the binding of PGG β-glucan, we next investigated the role that serum complement plays in binding. This was performed by (a) heat inactivating serum which non-specifically inactivates complement, and (b) specifically blocking C3, the complement protein central to the classical, alternate, and lectin complement pathways, with compstatin (38).

**Figure 3:**
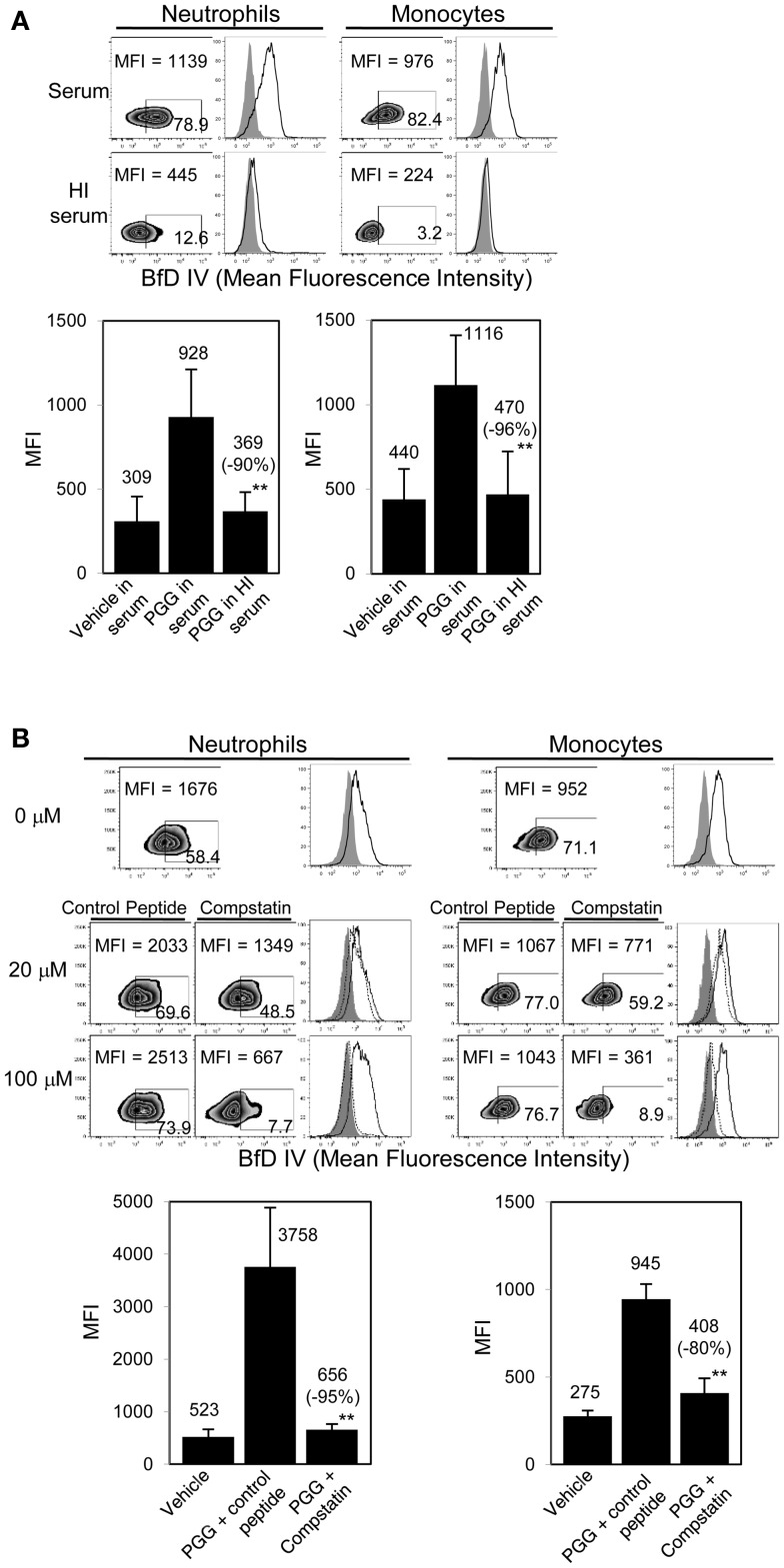
**Role of complement in binding of PGG β-glucan to human neutrophils and monocytes**. **(A)** Binding of PGG β-glucan to human neutrophils and monocytes in 10% serum (upper row) was compared to binding in 10% HI serum (lower row). Histograms show PGG β-glucan binding (solid) in comparison to vehicle-control (gray filled). Bar represents mean MFI from five donors with percentage of inhibition indicated in parenthesis. **p ≤ 0.05 compared to binding in serum. **(B)** Inhibitory effect of C3-inhibitor compstatin on PGG β-glucan binding in neutrophils and monocytes was measured and compared to that of control peptide-treated group. Histograms show PGG β-glucan binding in the presence of peptide control (solid) or compstatin (dotted) in comparison to vehicle-control (gray filled). Bar represents mean MFI with percentage of inhibition indicated in parenthesis. **p ≤ 0.05 compared to binding in control peptide. Data shown are representative of at least three independent experiments performed with cells obtained from different donors.

As shown in Figure 3A, binding of PGG β-glucan to both neutrophils and monocytes was reduced when the serum was HI. Based on MFI values from five separate donors, the percentage of inhibition of binding in HI serum was found to be 71–100% with an average inhibition of 90%, and in monocytes the inhibition range was 81–100% with an average of 96%. The data in Figure 3B further show that specifically blocking activation of C3 using varying concentrations of compstatin inhibited binding of PGG β-glucan in a concentration-dependent manner, with maximum inhibition observed at 100 μM. The control peptide had no effect on binding of PGG β-glucan on monocytes, but for reasons unknown, binding on neutrophils non-specifically increased in the presence of peptide control. Based on MFI values from three separate donors, the percentage of inhibition of binding by compstatin in neutrophils was 90–98% with an average inhibition of 95%, in monocytes the range was 62–92% with an average of 80%. Taken together, these data conclusively show that complement plays a critical role in binding of soluble PGG β-glucan to human neutrophils and monocytes.

### Serum, time, and temperature requirement for binding of PGG β-glucan to human neutrophils and monocytes is primarily at the ligand level and not at the receptor level

**Figure 4:**
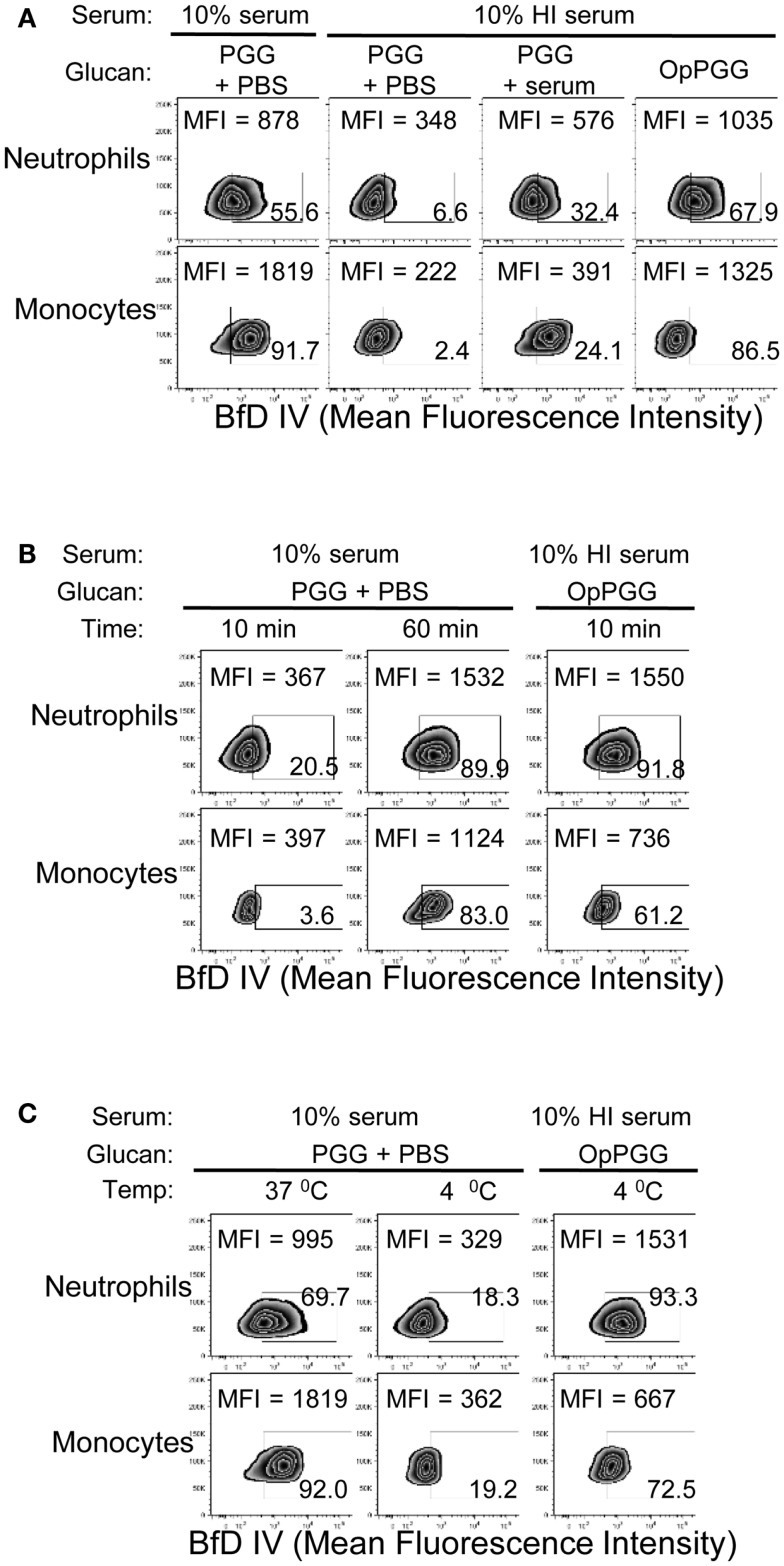
**Requirement of optimal percentage of serum, incubation time, and incubation temperature at the ligand level versus the cellular level**. **(A)** PGG β-glucan-treated with PBS (PGG + PBS), PGG β-glucan and serum added separately (PGG + serum), or serum pre-opsonized PGG β-glucan (OpPGG) were added at concentration 100 μg/mL to neutrophils or to PBMC, incubated for 1 h at 37°C in 10% serum or 10% HI serum, and binding was measured by flow cytometry. **(B)** Binding was measured at 37°C after incubating PGG or OpPGG with the cells for 10 min or 1 h in RPMI containing 10% serum or 10% HI serum. **(C)** Binding was measured at 4°C after incubating PGG or OpPGG with cells as described above. Data shown in the zebra plots with MFI and percentage of BfD IV positive population are representative of three independent experiments performed with cells obtained from three different donors.

The findings of optimal serum content, incubation time, and temperature, together with the critical requirement of complement for binding of PGG β-glucan, led us to hypothesize that PGG β-glucan is opsonized by complement proteins. To test this hypothesis, we first investigated whether the dependency on time, temperature, and serum is indeed at the ligand (i.e., PGG β-glucan) level and not at the receptor (i.e., CR3) level. To discern the influence of serum, time, and temperature on the ligand versus the receptor, we designed an experiment where the PGG β-glucan was pre-opsonized with serum at 37°C for 30 min, added to cells that were resuspended in media supplemented with HI serum (shown in Figure 4A as non-permissive condition for binding), and we then subsequently measured rescue of binding to the cells. As described in the methods section, the percentage of serum carried over along with the pre-opsonized PGG β-glucan was kept under 2%. Results in Figure 4A show that the pre-opsonized PGG β-glucan (OpPGG) was able to rescue binding on cells in HI serum. While the extent of rescued binding as measured by MFI and the percentage of cells positive for BfD IV staining was minimal for unopsonized PGG β-glucan (PGG) and PGG β-glucan plus serum added separately to the cells (PGG + serum), the binding obtained by pre-opsonized PGG β-glucan was comparable to that observed for cells cultured with PGG β-glucan in 10% serum. Interestingly, as shown in Figure 4B, the pre-opsonized PGG β-glucan was also able to bind to cells resuspended in HI serum within 10 min of incubation, which required 60 min for naïve PGG β-glucan in 10% serum. Furthermore, the pre-opsonized PGG β-glucan could even recover binding on cells resuspended in HI serum at 4°C (Figure 4C), a condition, which otherwise gave minimal binding. The efficacy of the pre-opsonization process for PGG β-glucan itself was also found to be dependent on time, and temperature: longer incubation time, and higher incubation temperature (physiologic, 37°C) of the PGG-serum pre-opsonization mixture, resulted in better rescue of binding to cells (Figure S3 in Supplementary Material).

These results demonstrate that serum, time, and temperature are critical factors for β-glucan binding to cells from the ligand, i.e., PGG β-glucan perspective, and do not appear to be relevant factors for CR3 modulation.

### Opsonization of soluble PGG β-glucan occurs by interaction of complement proteins with the β-glucan

After demonstrating the critical prerequisite of serum opsonization of PGG β-glucan in order for cells to bind the glucan, we further investigated our hypothesis of complement opsonization of β-glucan by determining the interaction between PGG β-glucan and one of the major complement opsonins, iC3b, which is also a CR3 receptor ligand.

**Figure 5:**
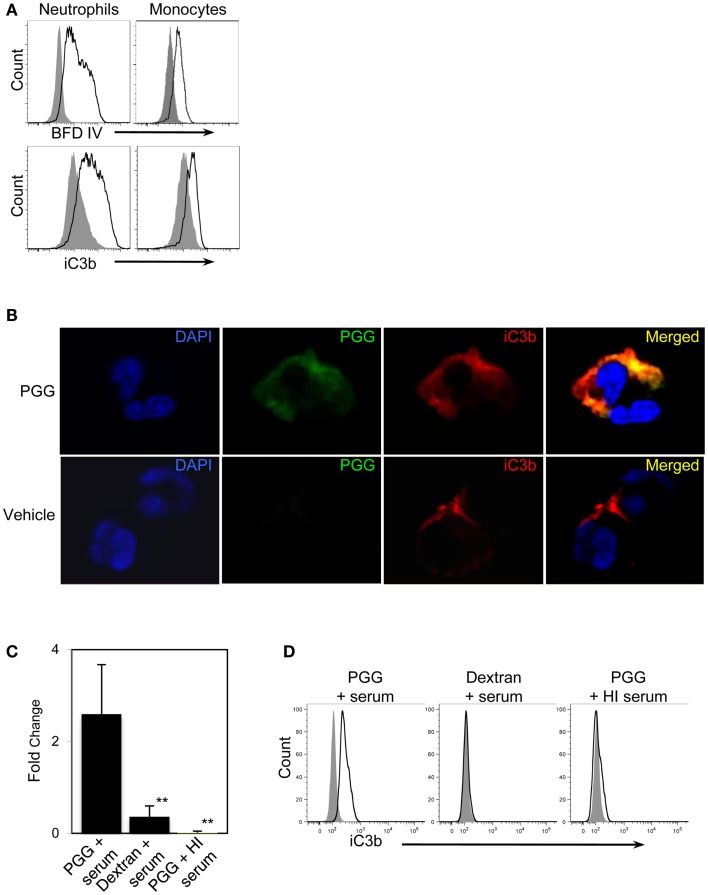
**Detection of the complement opsonin, iC3b on the surface of PGG β-glucan-bound cells**. **(A)** Fluorescence intensity of BfD IV- (upper row) and anti-iC3b-stained cells (lower row) treated with PGG β-glucan (solid) in comparison to that of the vehicle-treated control cells (gray filled). Data shown here are representative of three independent experiments. **(B)** Confocal microscopy images of surface staining of PGG β-glucan (middle panel, in green), iC3b (third panel from left, in red), or the merged image of PGG and iC3b (far right panel) on PGG β-glucan-treated (upper row), and vehicle-treated neutrophils (lower row). Neutrophil nuclei stained with DAPI is shown in blue. Shown here are representative data from two independent experiments. **(C)** Direct interaction of iC3b with immobilized PGG β-glucan in serum or HI serum was evaluated by ELISA. Dextran was used as control glucan. Bars represent mean fold change values from three independent experiments. **p ≤ 0.05 compared with PGG β-glucan-treated with serum. **(D)** iC3b present in the immunoprecipitated PGG β-glucan-serum complex was detected by Flow Cytometry. The histogram shows the comparison of iC3b detected on the beads pulled-down from the various glucan-serum mixtures (solid) in comparison to that from the vehicle-serum mixture (gray filled). Data shown are representative of three independent experiments.

First, we reasoned that if PGG β-glucan is being opsonized by iC3b, then this protein should be detected on the neutrophils and monocytes that are binding PGG β-glucan. Results shown in Figure 5A demonstrate increased staining of iC3b on monocytes and neutrophils incubated with PGG β-glucan in media containing 10% serum in comparison to iC3b staining levels on cells incubated in media containing 10% serum alone with no β-glucan present.

We next qualitatively determined by confocal microscopy whether the β-glucan and iC3b appear on close proximity on cells binding PGG β-glucan. Results presented in Figure 5B show the detection of β-glucan and iC3b on a neutrophil using BfD IV and a mAb against iC3b respectively; both fluorophores are visually brighter on the PGG β-glucan-treated cell versus the vehicle-treated cell. Merging of the BfD IV and iC3b mAb fluorescence emission signals clearly indicates localization of the bound PGG β-glucan and iC3b protein in very close or identical spatial positions on a neutrophil.

The results from confocal microscopy were further corroborated by evaluating the actual physical interaction of PGG β-glucan with iC3b. This was performed using (a) solid phase immunoassay system where the PGG β-glucan was immobilized to a solid phase, incubated with serum, and then the iC3b protein bound to the PGG β-glucan was detected by ELISA, and (b) immunoprecipitation where PGG β-glucan was immunoprecipitated in fluid-phase from a mixture of the PGG glucan incubated with serum using the BfD IV as the immunoprecipitating antibody, and subsequently subjecting the immunoprecipitated material to flow cytometric detection of co-immunoprecipitated iC3b protein. The results obtained from the ELISA showed that the fold increase of iC3b detected on wells with immobilized PGG β-glucan over that of the background was significantly higher than the fold increase on dextran-bound wells or wells with immobilized PGG β-glucan incubated in HI serum (Figure 5C). In the immunoprecipitation studies, iC3b protein was pulled-down along with the BfD IV-precipitated PGG β-glucan, while no iC3b was detected when the β-glucan was immunoprecipitated in HI serum. The absence of iC3b in the immunoprecipitated dextran-serum mixture indicated that the iC3b interaction was specific to PGG β-glucan (Figure 5D). Thus, these data provide evidence that soluble PGG β-glucan becomes opsonized when incubated with serum by interacting with one of the complement opsonins, iC3b, and that opsonization plays a critical role in binding of the PGG β-glucan to neutrophils and monocytes.

### Alternative pathway of complement activation is partially involved in opsonization of soluble PGG β-glucan

**Figure 6:**
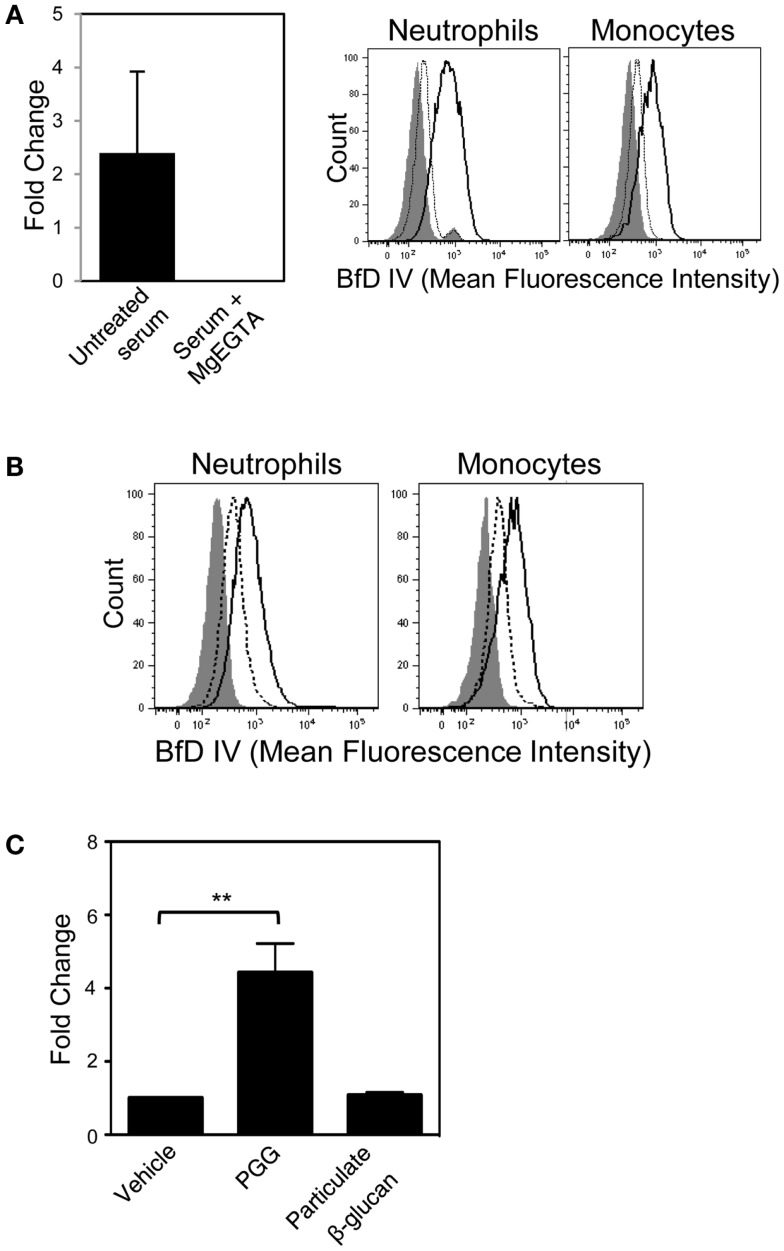
**Role of the alternative complement pathway in binding of PGG β-glucan to neutrophils and monocytes**. **(A)** The effect of MgEGTA-treated serum (Mg + EGTA) on iC3b deposition on immobilized PGG β-glucan was measured by ELISA (left), and on OpPGG preparation and subsequent binding to neutrophils and monocytes (right) was measured by flow cytometry. Histograms show binding of OpPGG prepared with untreated serum (solid), OpPGG prepared with MgEGTA-treated serum (dotted) and vehicle binding (gray filled) to neutrophils and monocytes in 10% HI serum. **(B)** The effect of anti-Factor D Ab, 166-32 on PGG β-glucan binding to neutrophils and monocytes was measured by flow cytometry. Histograms show cell binding to PGG β-glucan in the presence of isotype control (solid) or 166-32 mAb (dotted) in comparison to vehicle-control binding (gray filled). Data shown are representative of three independent experiments with serum from three different donors. **(C)** Activation of classical or lectin complement pathway as measured by C4a generation upon stimulation of WB with PGG or particulate β-glucan was determined by ELISA. Bar represent mean fold change values from three independent experiments. **p ≤ 0.05 compared to vehicle-treated WB.

In order to evaluate whether the soluble PGG β-glucan, like its particulate counterparts, requires alternative pathway of complement activation for opsonization, we first employed the approach of differential chelation of divalent cations that are critical for functioning of the alternative, classical, and lectin complement pathways. MgEGTA treatment of serum (addition of magnesium ions in an equimolar concentration to EGTA) allows optimal complement activation by the alternative pathway while completely inhibiting the calcium sensitive classical and/or lectin pathways (24, 25, 39–43). As shown in Figure 6A (left side), iC3b deposition on plate-bound β-glucan was completely inhibited in MgEGTA-treated serum. Binding to cells by PGG β-glucan pre-opsonized with either untreated, or MgEGTA-treated serum (and then resuspended in media containing HI serum) was also evaluated. Pre-opsonization of PGG β-glucan in untreated serum allowed binding to occur, while the rescue of binding was highly diminished when the β-glucan was reacted in MgEGTA-treated serum (Figure 6A, right side). The carried over EGTA did not affect binding of β-glucan on cells in serum (non-HI) indicating that the inhibition effect was specifically due to the abolished classical and/or lectin pathway of complement activity and not due to potential blocking of CR3 function (data not shown).

In order to further confirm the role of alternative complement pathway in opsonization and binding of PGG β-glucan, we investigated the effect of selectively blocking the alternative pathway on binding of PGG β-glucan. Factor D, the protease critical for functioning of alternative pathway was blocked using the anti-Factor D mAb, 166-32. Binding of PGG β-glucan to neutrophils and monocytes was evaluated using serum treated with either anti-Factor D or isotype control antibody. Interestingly, as shown in Figure 6B, blocking of the alternative pathway partially inhibited binding of the PGG β-glucan to both neutrophils and monocytes. Prior to evaluating MgEGTA- or 166-32-treated serum in binding studies with PGG β-glucan, their ability to selectively block either the EA-activated classical pathway or the CVF-activated alternative pathway was investigated using the SC5b-9 measurement kit. As shown in Figure S4A in Supplementary Material, MgEGTA treatment of the serum completely blocked the classical pathway, but allowed functioning of the CVF-activated alternative pathway. Likewise, the 166-32 mAb specifically blocked the CVF-activated alternative pathway but not the EA-activated classical pathway (Figure S4B in Supplementary Material).

These data, taken together demonstrate that calcium depletion had a more pronounced inhibition effect on opsonization and binding of PGG β-glucan. The opsonization and binding of PGG β-glucan was only partially affected by inhibiting alternative pathway activation. Binding did not occur in the absence of the classical or the lectin pathway, even when the alternative pathway was fully functional. The potential role of the classical or the lectin pathway in initiating the complement activation was further corroborated by measuring the levels of C4a, one of the complement proteins produced downstream of interaction of either the C1 protein with the immune complex (classical pathway), or the MBL with the mannose containing pathogen surfaces (lectin pathway). As expected, the results shown in Figure 6C demonstrate that incubation of 10 μg/mL of PGG glucan with WB at 37°C for 30 min was sufficient to activate complement and produce significant levels of C4a in the plasma. In contrast, the particulate glucan, the prototype activator of alternative pathway of complement activation did not produce any C4a in the serum.

## Discussion

In order for the particulate and soluble yeast β-glucans to be used for therapeutic purposes, an understanding of their interactions with human neutrophils and monocytes is required. In this study, we investigated the binding characteristics of *Saccharomyces cerevisiae*-derived, relatively pure and analytically well-characterized soluble PGG β-glucan to human neutrophils and monocytes. This investigation demonstrated that CR3 is the main receptor on human neutrophils and monocytes for PGG β-glucan and that the binding of soluble PGG β-glucan to CR3 is complement-dependent.

The role of complement proteins in the binding of PGG β-glucan to human neutrophils and monocytes was elucidated in several different ways. Dependency of binding on serum (Figure 2A) and abrogation of binding upon heat-inactivation of serum (Figure 3A) clearly implicated complement. Significant inhibition of PGG β-glucan binding in the presence of compstatin-treated serum further validated the role of complement (Figure 3B). As compstatin, by selectively binding to C3, inhibits the cleavage of C3 into its activated form C3b, the data indicated that conversion of C3 into C3b was the critical initial step required for binding of PGG β-glucan to cells (44).

As the process of activation of complement proteins in the serum requires optimal reaction time and temperature, it was not surprising that the binding of PGG β-glucan increased as a function of incubation time and temperature during incubation with the cells (Figures 2B,C). Since PGG β-glucan did not bind to cells at 4°C and the amount of surface-bound PGG β-glucan increased over the incubation time, it is unlikely that internalization of PGG β-glucan occurred during the tested time-frame. The pre-opsonization step with PGG β-glucan allowed binding to occur on the cells in the HI serum at 4°C in 10–30 min, which are often the conditions used to measure ligand binding to cells (Figure 4). Internalization of the pre-opsonized PGG β-glucan need to be investigated by performing binding studies in WB and extending the incubation time beyond 2 h.

In contrast to our findings, earlier studies demonstrated binding of a soluble low molecular weight β-glucan to human neutrophils in the presence of low concentrations of serum or at 4°C (7, 8). Differences between our findings and those previously reported could be due to several reasons, including differences in the sugar, molecular weight, contaminants, or other structural aspects that drive activity. Since the previous studies used FITC or radioactive labeling to measure binding, it is also possible that these labeling techniques may have made detection sensitive enough to see small amounts of β-glucan bound to cells; in addition, the effects of further increasing serum, time, or temperature were never explored. In contrast to our findings, another published study using DTAF-labeled PGG β-glucan demonstrated no binding to human neutrophils (11). This discrepancy is likely due to the binding studies being performed using concentrations of β-glucan that were too low (0.5–2 μg/mL) to detect binding.

Complement-mediated opsonization involves a covalent bond formation between the thioester group in C3b, the active fragment of C3 with the hydroxyl groups of carbohydrates or amine or hydroxyl groups of proteins present in immune complexes and on the surface of pathogens (45, 46). After C3b deposits on a substrate, it can be proteolyzed to iC3b and then to C3d. Complement fragments, specifically, C3b and iC3b bound to β-glucan have been detected on particulate zymosan and intact yeast (1, 26, 47). In this study, evidence for opsonization of soluble PGG β-glucan was obtained by detection of iC3b complement protein attached to cell surface-bound PGG β-glucan and to purified PGG β-glucan itself using a mAb specific for iC3b (Figures 5A–D). Although complement activation and opsonization of immune complexes in fluid-phase is well-described (48, 49), the result of opsonization of soluble β-glucan, a fungal PAMP, is novel. As noted, the attachment of C3b to a pathogen can be via an ester bond on hydroxylated targets, and it is reasonable that C3b can bind to PGG β-glucan *via* covalent interaction with the abundant hydroxyl groups present on the carbohydrate (50).

The use of natural ligands for CR3 and monoclonal Abs that bind to and block discrete regions of CR3 have allowed for a greater understanding of the specific interactions of β-glucans with CR3. Ross and colleagues demonstrated previously that blocking Abs to the CR3 I-domain and lectin domain inhibited opsonized yeast activation of human phagocytes; the opsonized yeast-CR3 interaction was shown to occur via binding of fixed iC3b to the I-domain as well as by binding of yeast β-glucan to the lectin domain, giving rise to the term “dual-ligation” receptor (2). In our study, similar to the finding with opsonized yeast, inhibition of binding of PGG β-glucan to human neutrophils and monocytes was observed when both the I- and lectin-domains of CR3 were blocked (Figure 1B). However, maximum inhibition of binding was consistently achieved by blocking the CD18 chain along with the CD11b chain. These data indicate a probable role of the CD18 chain in allowing the CD11b chain to attain a conformation that is conducive to binding both iC3b and β-glucan present in opsonized PGG β-glucan. This observation is supported by findings of previous studies where the CD18 subunit has been shown to be involved in the “inside-out” signaling that brings about conformational changes within the ligand binding I-domain (51). Furthermore, binding of iC3b to CR3 was also demonstrated to be significantly inhibited by mAbs blocking the CD18 chain (52, 53).

Although Dectin-1 is predominantly a receptor for unopsonized particulate glucans, some studies have shown binding of opsonized zymosan to Dectin-1 based on inhibition of binding in the presence of soluble laminarin (3, 4, 54, 55). Laminarin, a seaweed glucan, has routinely been used as a Dectin-1 antagonist, however laminarin has also been shown to be a ligand for CR3 (7, 8). Our attempts at inhibiting binding of PGG β-glucan in the presence of excess laminarin produced inconclusive results (unpublished observations).

**Figure 7:**
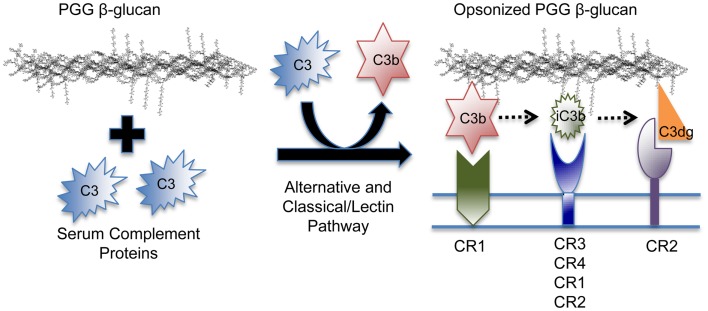
**Model of the binding mechanism of PGG β-glucan to complement receptors on human immune cells**. PGG β-glucan has the ability to activate complement and to be opsonized by C3b, which can then be degraded to iC3b, and C3dg, which remain covalently bound. Since the different complement fragments preferentially bind to specific complement receptors, the opsonized PGG β-glucan has the potential to bind to CR1 via C3b, or CR3 via iC3b, or CR2 via C3dg. This study demonstrates that complement opsonization of soluble yeast β-glucan makes it amenable for recognition by several complement receptors on human innate and possibly, adaptive immune cells.

In addition to C3b and iC3b, it is quite likely that other C3 fragments, including C3dg, the proteolytic breakdown fragment of iC3b, is also covalently bound to PGG β-glucan. Different fragments show preferential binding to specific complement receptors, i.e., C3b binds specifically to CR1, C3dg to CR2, and iC3b is recognized by all complement receptors, but binds more avidly to CR3 and CR4 (56). Therefore, PGG β-glucan opsonized with different C3 degradation fragments has the potential of being recognized by these other complement receptors expressed on both innate and adaptive immune cells (Figure 7). It will be interesting to investigate the role of CR1 in particular, because it is a co-factor known to play a role in the conversion of C3b to iC3b and to C3dg, and also because earlier studies have shown the co-operativity between CR1 and CR3 in allowing sustained binding of iC3b-opsonized pathogens to CR3 (1, 57–59).

In our study, opsonization and binding of PGG β-glucan was completely inhibited by calcium chelation, and was not rescued in serum treated with MgEGTA. However, specifically blocking only the alternative pathway by Factor D antibody partially inhibited binding of PGG β-glucan to both neutrophils and monocytes. Furthermore, PGG β-glucan was also able to produce the classical and/or the lectin pathway-specific C4a complement protein when incubated with WB. These data suggest that the alternative pathway plays a role in amplifying the complement activation mediated by PGG β-glucan (Figure 6). It is quite likely that unlike the particulate form of β-glucan, PGG β-glucan initially activates the classical or the lectin pathway, and then the nascent C3b feeds over into the alternative pathway to further amplify complement activation. Further studies are required to confirm whether the lectin or the classical pathway is activated by PGG β-glucan.

In summary, our study demonstrates the critical role of complement in binding soluble yeast β-glucan to human neutrophils and monocytes. The discovery of the ability of soluble yeast β-glucan to activate complement and become opsonized expands the horizon for its immunomodulatory activities as it could be recognized by several complement receptors on a repertoire of human innate and adaptive immune cells. It is important to understand the binding characteristics of a soluble pharmaceutical-grade yeast β-glucan preparation to human immune cells as several clinical studies for different therapeutic applications of β-glucan are either underway or being developed (http://clinicaltrials.gov/ct2/results?term=beta+glucan). The results of this investigation could have implications on the design of basic research as well as clinical research studies based on the clinical application of soluble yeast β-glucan.

## Conflict of Interest Statement

The authors, Nandita Bose, Anissa S. H. Chan, Xiaohong Qiu, Richard M. Walsh, Kathleen E. Ertelt, Adria Bykowski Jonas, Keith B. Gorden, Lindsay R. Wurst, Michael E. Danielson, Andrew S. Magee, and Myra L. Patchen are employes of Biothera and are beneficiaries of the Biothera employe stock plan. Faimola Guerrero, Carolyn M. Maristany, Christine M. Dudney, and Natalie Elmasry are former employes. John P. Vasilakos is a former employe of Biothera, is currently a consultant to Biothera and has equity in the company. The authors have no relevant affiliations or financial involvement with any organization or entity with a financial interest in or financial conflict with the subject matter or materials discussed in the manuscript apart from those disclosed.

## Supplementary Material

The Supplementary Material for this article can be found online at: http://www.frontiersin.org/Molecular_Innate_Immunity/10.3389/fimmu.2013.00230/abstract

Supplementary Figure S1 **Evaluation of donor variability in concentration-dependent binding of PGG β-glucan**. Binding of increasing concentrations of PGG β-glucan (0, 10, 25, 100, 200, and 400 μg/mL) to neutrophils (left) and monocytes (right) was determined by flow cytometry as described in the Section “Materials and Methods”. The graphical representation shows the MFI of PGG β-glucan-bound neutrophils and monocytes; each symbol (■ for neutrophils and • for monocytes) represents one individual from five separate experiments. The average MFI obtained at each of the concentrations is indicated by a horizontal bar.

Supplementary Figure S2 **Evaluation of donor variability in serum-dependent binding of PGG β-glucan**. Binding of 100 μg/mL PGG β-glucan to neutrophils (left) and monocytes (right) at 2, 5, 10, 20, and 50% of serum or 10% heat-inactivated (HI) serum was determined by flow cytometry as described in the Section “Materials and Methods”. The graphical representation shows the MFI of PGG β-glucan-bound neutrophils and monocytes; each symbol (■ for neutrophils and • for monocytes) represents one individual from five separate experiments. The average MFI obtained at each of the serum concentrations is indicated by a horizontal bar.

Supplementary Figure S3 **Requirement of optimal incubation time and incubation temperature for pre-opsonization of PGG β-glucan in neutrophils**. The optimal temperature **(A)** and time **(B)** for OpPGG preparation and subsequent binding to isolated human neutrophils in HI serum at 37°C for 1 h was evaluated by flow cytometry. The MFI and percentage of β-glucan-treated, BfD IV positive cells are indicated in the zebra plots.

Supplementary Figure S4 **Evaluation of different serum treatments on the prototypical activators of classical and alternative pathways of complement activation**. The ability of **(A)** MgEGTA to block EA-activated CP alone and **(B)** anti-Factor D antibody, 166-32 to block CVF-activated AP was confirmed by fluid-phase SC5b-9 ELISA.
