# Supplementary figures and images for: Binding of Soluble Yeast β-Glucan to Human Neutrophils and Monocytes is Complement-Dependent
Source: Front Immunol. 2013 Aug 12;4:230. doi: 10.3389/fimmu.2013.00230 (PMC3740326; doi:10.3389/fimmu.2013.00230)

Supplementary Figure 1

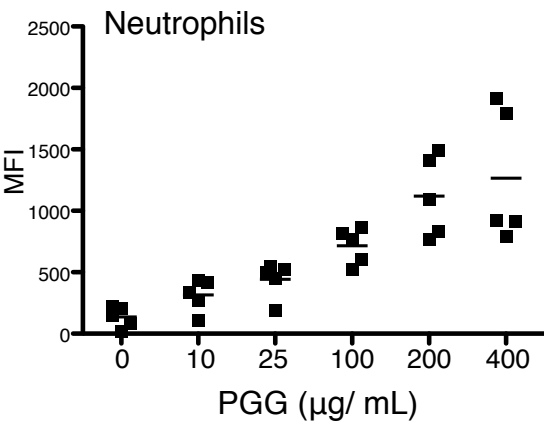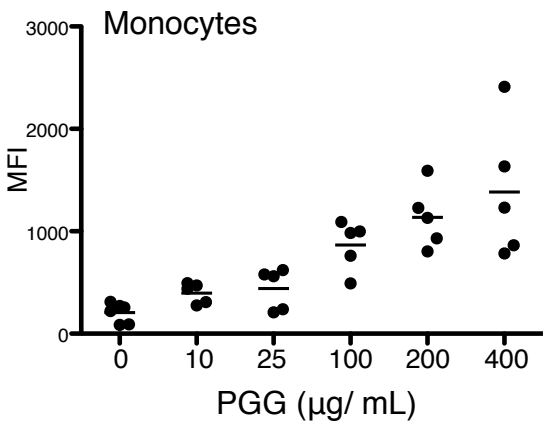

Supplementary Figure 2

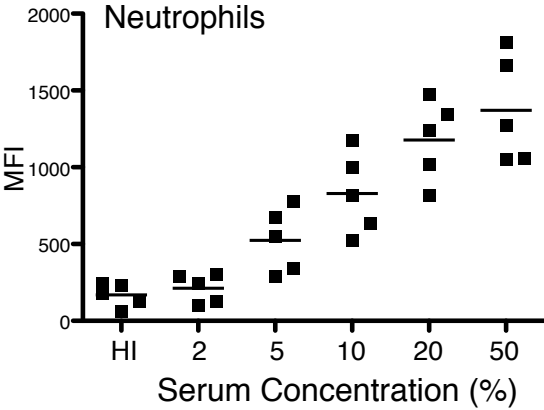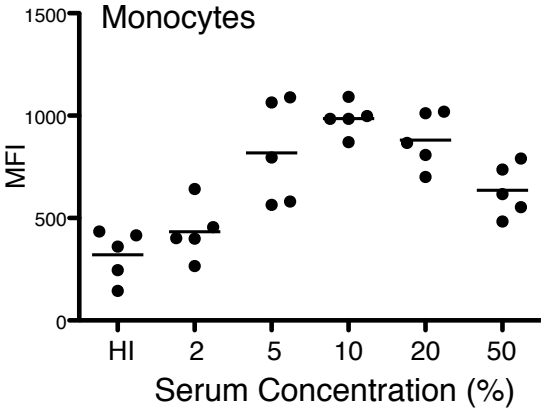

Supplementary Figure 3

A

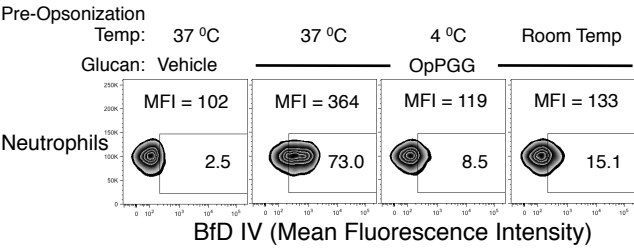

B

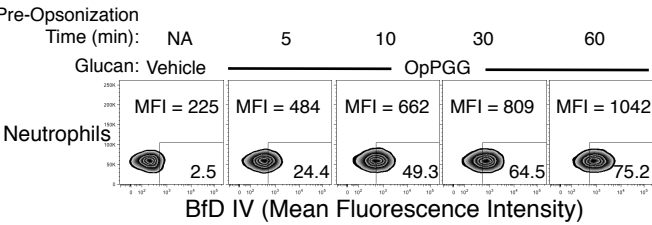

Supplementary Figure 4

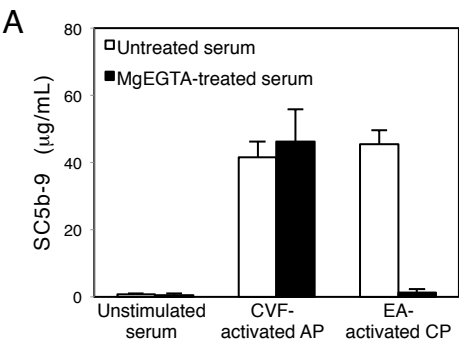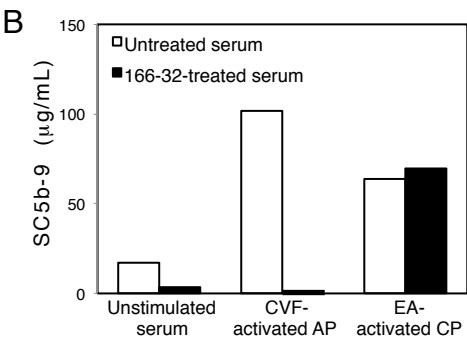

Supplement: Supplementary Figure S1 — Evaluation of donor variability in concentration-dependent binding of PGG β-glucan. Binding of increasing concentrations of PGG β-glucan (0, 10, 25, 100, 200, and 400 μg/mL) to neutrophils (left) and monocytes (right) was determined by flow cytometry as described in the Section “Materials and Methods”. The graphical representation shows the MFI of PGG β-glucan-bound neutrophils and monocytes; each symbol (■ for neutrophils and • for monocytes) represents one individual from five separate experiments. The average MFI obtained at each of the concentrations is indicated by a horizontal bar. [file 54728_Bose_Presentation1.PDF]
